# Classifying Human Leg Motions with Uniaxial Piezoelectric Gyroscopes

**DOI:** 10.3390/s91108508

**Published:** 2009-10-27

**Authors:** Orkun Tunçel, Kerem Altun, Billur Barshan

**Affiliations:** Department of Electrical and Electronics Engineering, Bilkent University, Bilkent 06800 Ankara, Turkey; E-Mails: orkun@ee.bilkent.edu.tr (O.T.); kaltun@ee.bilkent.edu.tr (K.A.)

**Keywords:** gyroscope, inertial sensors, motion classification, Bayesian decision making, rule-based algorithm, least-squares method, *k*-nearest neighbor, dynamic time warping, support vector machines, artificial neural networks

## Abstract

This paper provides a comparative study on the different techniques of classifying human leg motions that are performed using two low-cost uniaxial piezoelectric gyroscopes worn on the leg. A number of feature sets, extracted from the raw inertial sensor data in different ways, are used in the classification process. The classification techniques implemented and compared in this study are: Bayesian decision making (BDM), a rule-based algorithm (RBA) or decision tree, least-squares method (LSM), *k*-nearest neighbor algorithm (*k*-NN), dynamic time warping (DTW), support vector machines (SVM), and artificial neural networks (ANN). A performance comparison of these classification techniques is provided in terms of their correct differentiation rates, confusion matrices, computational cost, and training and storage requirements. Three different cross-validation techniques are employed to validate the classifiers. The results indicate that BDM, in general, results in the highest correct classification rate with relatively small computational cost.

## 1. Introduction

In this paper, we use inertial sensors to classify movements of the human leg and present the results of a comparative study for this purpose. Inertial sensors are self-contained, nonradiating, nonjammable, dead-reckoning devices that provide dynamic motion information through direct measurements. To effectively use the information from such sensors, however, it is essential to accurately describe, interpret and classify their outputs. The gyroscope, a type of inertial sensor, provides angular *rate* information around an axis of sensitivity. Similarly, an accelerometer provides linear or angular velocity rate information. Although this rate information is reliable over long periods of time, it must be integrated to provide absolute measurements of orientation, position and velocity. Thus, even very small errors in the rate information provided by inertial sensors cause an unbounded growth in the error of integrated measurements. As a consequence, the outputs of inertial sensors are characterized by position errors that grow with time and distance unboundedly. One way to overcome this problem is to periodically reset the output of inertial sensors with other absolute sensing mechanisms, therefore to eliminate this accumulated error. Thus, techniques of fusing inertial sensor data with other sensors such as GPSs, vision systems, and magnetometers have been widely adopted [1–3].

For several decades, inertial sensors have been used in various applications such as for navigation of aircraft [4–6], ships, land vehicles and robots [7–9], for state estimation and dynamic modeling of legged robots [10, 11], in the automotive industry, for shock and vibration analysis, and in telesurgery [12, 13].

Inertial sensing systems have become easy to design and carry as the size and cost of inertial sensors have decreased considerably with the rapid development of micro electro-mechanical systems (MEMS) [14]. Small, lightweight, low-cost miniature inertial sensors (e.g., gyroscopes, accelerometers, inclinometers or tilt sensors) are increasingly commercially available. Although a considerable improvement over past systems, they clearly provide substantially less accurate position information than highly accurate inertial systems such as those used in aerospace applications. Some of these devices are sensitive around a single axis; others are multi-axial (usually two- or three-axial). For low-cost applications that utilize these devices, gyro calibration, which is provided for high-end commercial gyros by the manufacturer is still a necessary and complicated procedure (requiring an accurate variable-speed turntable). Accelerometer-based systems are more commonly adopted because accelerometers are easily calibrated by gravity.

The developments in the MEMS area have opened up new possibilities for the use of inertial sensors, one of them being human activity monitoring, recognition, and classification through body-worn sensors. This in turn has a broad range of applications in observing the elderly remotely by personal alarm systems [15], detecting and classifying falls [16–18], medical diagnosis and treatment [19], home-based rehabilitation and physical therapy [20], monitoring children remotely at home or in school, ergonomics [21], sports science [22], ballet and other forms of dance [23], animation, film making, computer games [24, 25], professional simulators, virtual reality, and motion compensation and stabilization of equipment. References [26–29] and [30] provide comprehensive surveys on the use of inertial sensors in some of these areas.

The most commonly used approach in human activity recognition and classification employs vision-based systems with single or multiple video cameras [31–34]. For example, although the gesture recognition problem has been well studied in computer vision [35], much less research has been done in this area using body-worn inertial sensors [36, 37]. The use of camera systems may be acceptable and practical when activities are confined to a limited area (such as certain parts of a house or office environment) and when the environment is well illuminated. However, when the activity involves going from place to place (such as riding in or on a vehicle, traveling, going shopping, going outdoors, etc.), camera systems are not so convenient. Furthermore, camera systems interfere considerably with the privacy of the people involved and supply additional, unnecessary information. In addition to monitoring activities, they provide redundant information about the surroundings, other people in the area, and appearance, facial expressions, body language, and preferences of the person(s) involved.

Miniature inertial sensors can be flexibly used inside or behind objects without occlusion effects. This is a major advantage over visual motion capture systems that require free line-of-sight. When a single camera is used, the 3-D scene is projected onto a 2-D one, with significant information loss. Points of interest need to be pre-identified by placing special, visible markers such as light-emitting diodes (LEDs) on the human body. Some points of interest may be occluded or shadowed by human body parts or objects in the surroundings. This is circumvented by positioning multiple camera systems in the environment and using several 2-D projections to reconstruct the 3-D scene. Each camera needs to be calibrated individually. Another major disadvantage of using camera systems is that the cost of processing and storing images and video recordings is much higher than those of 1-D signals. 1-D signals acquired from multiple axes of inertial sensors can directly provide the required information in 3-D.

The use of camera systems and inertial sensors are two inherently different approaches that are by no means exclusive and can be used in a complementary fashion in many situations. In a number of studies, accelerometers are used in conjunction with video camera systems, considered as a comparison basis [38–43]. In other studies, visual sensors are not only used in a supplementary fashion or as a reference, but their data is actually integrated or fused with the inertial data [2, 44]. The fusion of visual and inertial sensor outputs has attracted considerable attention recently, due to their robust performance and potentially wide application [45, 46]. These two sensing modalities have complementary characteristics and can cover the limitations and deficiencies of each other: Inertial sensors have large measurement uncertainty in slow motion and lower relative uncertainty at high velocities. They can measure very large velocities and accelerations. On the other hand, cameras can track features very accurately at low velocities. With increasing velocity, tracking accuracy decreases because the resolution must be reduced to accommodate a larger tracking window for the same pixel size and a larger tracking velocity.

The work done on activity recognition through the use of body-worn inertial sensors until now is of limited scope, and mostly unsystematic in nature. The research undertaken by different parties has not been coordinated and exhibits a piece-wise collection of results, difficult to synthesize into the kind of broader understanding that is necessary to make substantial progress. Most previous studies distinguish between sitting, lying and standing [15, 38–40, 43, 47–50] as these postures are relatively easy to detect using the static component of acceleration. Distinguishing between walking, ascending and descending stairs has also been performed [47, 48, 50], although not as successfully as detecting postures. The signal processing and motion detection techniques employed, the configuration, number, and type of sensors differ widely among the studies, from using a single accelerometer [15, 51, 52] to as many as twelve [53] on different parts of the body. Although gyroscopes can provide valuable rotational information in 3-D, in most studies accelerometers are preferred over gyroscopes due to their ease of calibration. To the best of our knowledge, guidance on finding the optimal configuration, number, and type of sensors does not exist [47]. Usually, some configuration and some modality of sensors is chosen without strong justification, and empirical results are presented. Processing of the acquired signals and motion detection techniques are often also ad hoc and relatively unsophisticated. Furthermore, the available literature viewed as a whole is rather fragmented and incongruent, and the results are not directly comparable with each other; it is more like a scattered set of isolated results rather than a body of knowledge that builds on earlier work. A unified and systematic treatment of the subject is desirable.

In this work, we use small, low-cost gyroscopes positioned on the human leg to classify leg motions. The motivation behind investigating the differentiation of leg motions is its potential applications in physical therapy and home-based rehabilitation. For example, a patient with paralysis may be given certain exercises to do regularly, and inertial sensors can be used remotely to assess which exercise the patient is doing and whether he is doing it properly. We provide a systematic comparison between various classification techniques used for motion recognition based on the same data set. The comparison is in terms of the successful differentiation rates, confusion matrices, and computational requirements of the techniques.

The organization of this paper is as follows: In Section 2., we introduce the motions classified in this study and describe the experimental methodology. Feature selection and reduction process is the topic of Section 3. In Section 4., we review the classification methods used in this study. In Section 5., we present the experimental results and compare the computational costs of the methods. We also provide a brief discussion on the selection of classification techniques and their trade-offs. Section 6. briefly addresses the potential application areas of miniature inertial sensors in motion classification. In Section 7., we draw our conclusions and provide possible directions for future work.

## 2. Classified Leg Motions and Experimental Methodology

**Figure 1.**
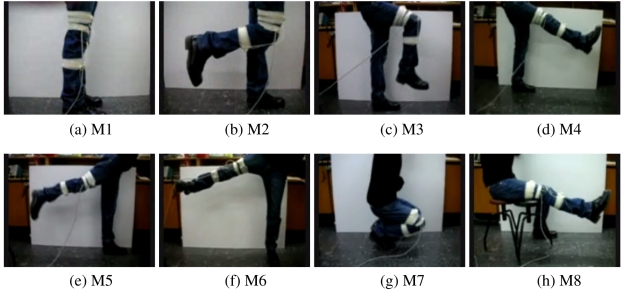
Eight different leg motions.

Eight different leg motions are classified using two single-axis gyroscopes that are placed on the subject's right leg. Photos taken while performing the motions are shown in Figure 1. Throughout the motions listed below, the subject's left foot stays on the ground. The motions are:

M1: standing without moving the legs (Figure 1(a)),
M2: moving only the lower part of right leg to the back (Figure 1(b)),
M3: moving both the lower and the upper part of the right leg to the front while bending the knee (Figure 1(c)),
M4: moving the right leg forward without bending the knee (Figure 1(d)),
M5: moving the right leg backward without bending the knee (Figure 1(e)),
M6: opening the right leg to the right side of the body without bending the knee (Figure 1(f)),
M7: squatting, moving both the upper and the lower leg (Figure 1(g)),
M8: moving only the lower part of the right leg upward while sitting on a stool (Figure 1(h)).

**Figure 2.**
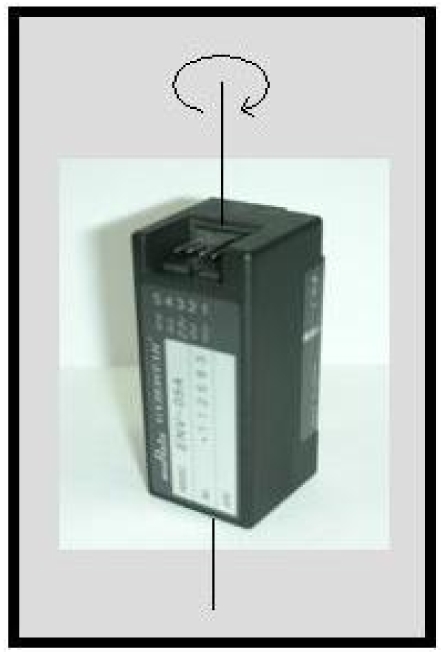
Murata Gyrostar ENV-05A.

The two gyroscopes used are Gyrostar ENV-05A piezoelectric vibratory gyroscopes manufactured by Murata (Figure 2). The Gyrostar is a small, relatively inexpensive piezoelectric gyro originally developed for the automobile market and active suspension systems [54]. The main application of the Gyrostar has been in helping car navigation systems to keep track of turns when, for short durations, the vehicle is out of contact with reference points derived from the additional sensors. It consists of a triangular prism made of a substance called “Elinvar”, on each vertical face of which a piezoelectric transducer is placed. Excitation of one transducer perpendicular to its face at about 8 kHz, causes vibrations to be picked up by the other two transducers. If the sensor remains still or moves in a straight line the signals produced by the pick-up transducers are exactly equal. If the prism is rotated around its principal axis, Coriolis forces proportionate to the rate of rotation are created.

This device operates with a DC supply voltage between eight and 13.5 V and converts angular velocity information to an analog DC voltage at its output [55]. The output voltage is proportional to the angular velocity of the device around its principal axis and varies between 0.5 and 4.5 V. The maximum rate that can be measured with the Gyrostar is ±90°/sec. An angular velocity of zero (no motion) corresponds to a voltage output of 2.5 V. At the maximum angular velocities of +90°/sec and −90°/sec, the output voltages become 4.5 V and 0.5 V, respectively. If the angular velocity is larger than the maximum value (±90°/sec), saturation occurs at the corresponding voltage level (0.5 or 4.5 V) so that the rate and the orientation information become erroneous and need to be reset.

**Figure 3.**
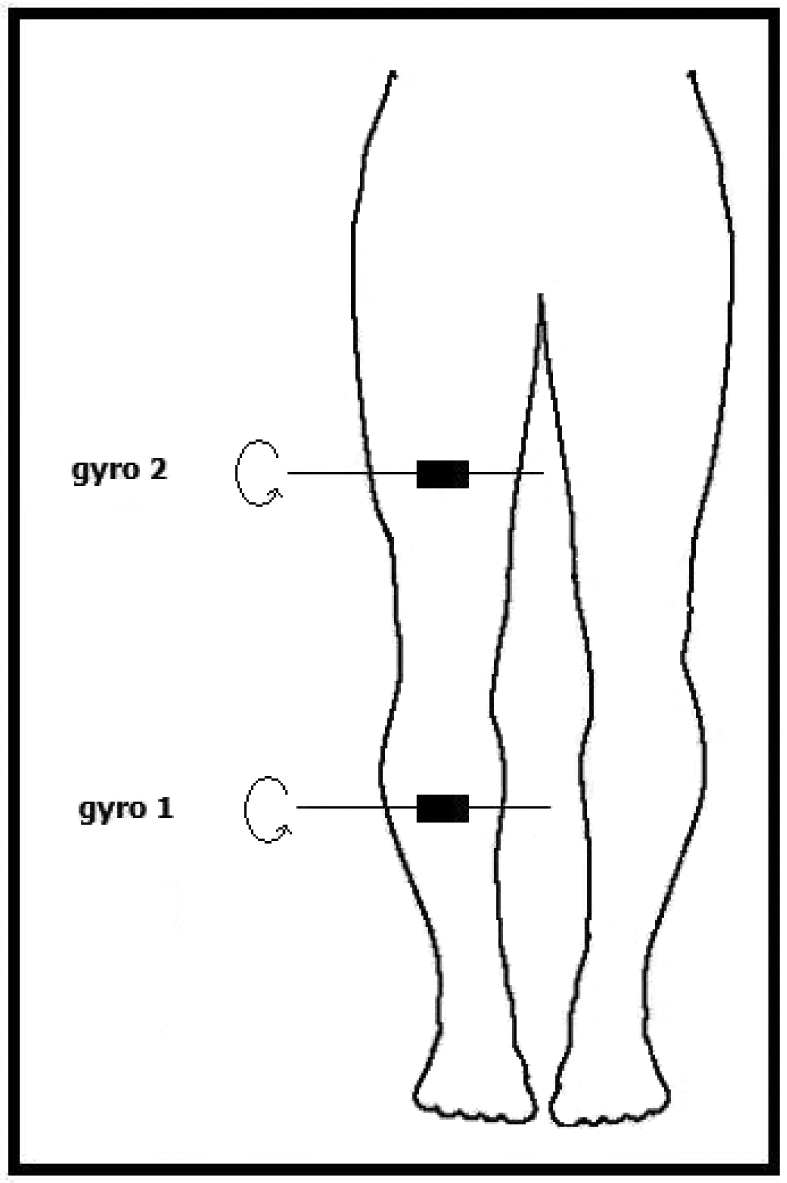
Position of the two gyroscopes on the human leg (body figure adopted from http://www.answers.com/body breadths).

Because these devices are sensitive to rotations around a single axis, the positioning of these sensors should be done by taking their sensitivity axis into account. One of the gyroscopes is placed 17 cm above and the other one 15 cm below the right knee of the subject, as illustrated in Figure 3. The sensors' sensitivity axes are placed parallel to the ground and to the front of the body. In this way, the highest number of different motions can be detected.

**Figure 4.**
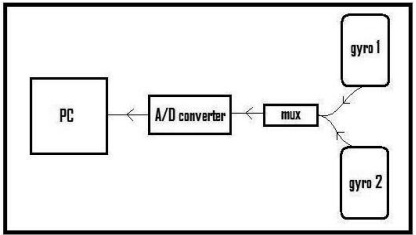
Block diagram of the experimental setup.

The block diagram of the experimental setup is given in Figure 4. The experimental setup comprises two piezoelectric gyroscopes for sensing the leg motions, a multiplexer to multiplex the signals of the two gyros, an eight-bit analog-to-digital (A/D) converter with a sampling frequency of 2,668 Hz, and a PC. Data acquired by the A/D converter is recorded on the PC through the parallel port of the computer with a simple interface program written in Turbo C++. After acquiring and storing this data, the signals are downsampled by 23 to obtain 116 Hz digital signals. Sensor signal processing is done using MATLAB.

In a laboratory environment, a male subject performs the above eight motions. A motion is performed repetitively for approximately 72 seconds, and the motion itself takes about five to seven seconds, for approximately 10 motions per 72-second interval. The same motion is repeated for another seven 72-second intervals. The subject then performs the next motion for the total of eight 72-second intervals. When he has performed all eight motions, the total signal duration per leg motion then, is approximately 576 (= 8 × 72) seconds.

**Figure 5.**
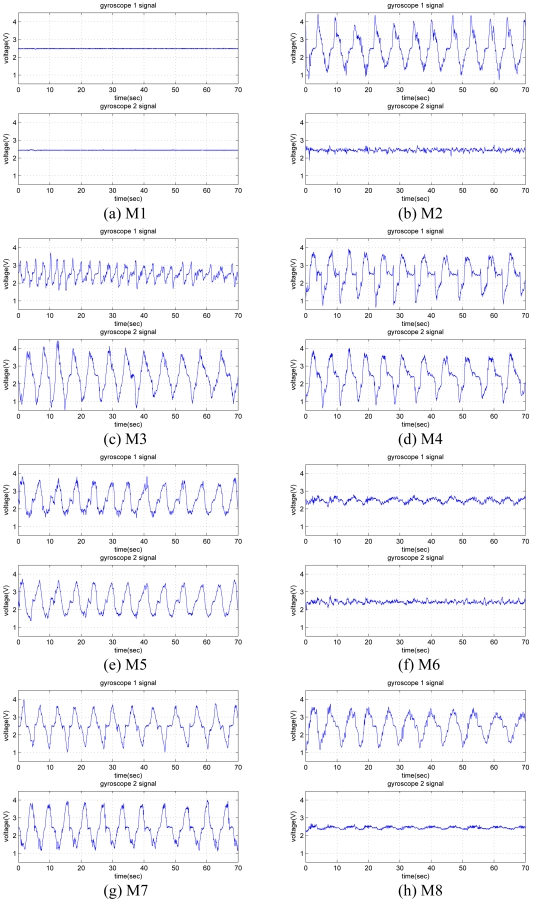
Signals of the two gyroscopes (gyro 1 and gyro 2) for the eight different leg motions.

The last 70 seconds of each 72-second signal is used and divided into 10-second time windows. Hence, while acquiring signals for each motion, a total of 56 (= 7 × 8) ten-second windows are recorded from each gyroscope. As there are two gyroscopes, 112 (= 56 × 2) signals are available for each motion. Sample gyroscope signals for eight different leg motions are given in Figure 5, where the quasi-periodic nature of the signals can be observed.

## 3. Feature Extraction and Reduction

After acquiring the signals as described above, a discrete-time sequence of *N_s_* elements that can be represented as an *N_s_* × 1 vector **s** = [*s*_1_, *s*_2_, …, *s_Ns_*]*^T^* is obtained. For the 10-second time windows and the 116 Hz sampling rate, *N_s_* = 1, 160. We considered using features such as the minimum and maximum values, the mean value, variance, skewness, kurtosis, autocorrelation sequence, cross-correlation sequence, total energy, peaks of the discrete Fourier transform (DFT) with the corresponding frequencies, and the discrete cosine transform (DCT) coefficients of **s**. DCT is a transformation technique widely used in image processing that transforms the data into the form of the sum of cosine functions [56]. The features used are calculated as follows, with an explanation below of why we chose our final set of features:

(1) $$\begin{array}{c} \text{mean}(\text{s})=\mu_{\text{s}}=E\{\text{s}\}=\frac{1}{N_{s}}\sum_{i=1}^{N_{s}}s_{i}\\ \text{variance}(\text{s})=\sigma^{2}=E\{{\left(\text{s}-\mu_{\text{s}}\right)}^{2}\}=\frac{1}{N_{s}}\sum_{i=1}^{N_{s}}{\left(s_{i}-\mu_{\text{s}}\right)}^{2}\\ \text{skewness}(\text{s})=\frac{E\{{\left(\text{s}-\mu_{\text{s}}\right)}^{3}\}}{\sigma^{3}}=\frac{1}{N_{s}\sigma^{3}}\sum_{i=1}^{N_{s}}{\left(s_{i}-\mu_{\text{s}}\right)}^{3}\\ \text{kurtosis}(\text{s})=\frac{E\{{\left(\text{s}-\mu_{\text{s}}\right)}^{4}\}}{\sigma^{4}}=\frac{1}{N_{s}\sigma^{4}}\sum_{i=1}^{N_{s}}{\left(s_{i}-\mu_{\text{s}}\right)}^{4}\\ \text{autocorrelation}:R_{\text{ss}}(k)=\frac{1}{N_{s}-\Delta }\sum_{i=0}^{N_{s}-\Delta -1}\left(s_{i}-\mu_{\text{s}})(s_{i-\Delta }-\mu_{\text{s}}\right)\;\Delta =0,1,\ldots ,N_{s}-1\\ \text{crosscorrelation}:R_{\text{su}}(\Delta )=\frac{1}{N_{s}-\Delta }\sum_{i=0}^{N_{s}-\Delta -1}\left(s_{i}-\mu_{\text{s}})(u_{i-\Delta }-\mu_{u}\right)\;\Delta =-N_{s}+1,\ldots ,0,\ldots ,N_{s}-1\\ \text{DFT}:S_{\text{DFT}}(k)=\sum_{i=0}^{N_{s}-1}s_{i}e^{-\frac{j2\pi ki}{N_{s}}}k=0,1,\ldots ,N_{s}-1\\ \text{DCT}:S_{\text{DCT}}(k)=\alpha (k)\sum_{i=0}^{N_{s}-1}s_{i}\cos\left[\;\frac{\pi (2i+1)}{2N_{s}}\right]\;k=0,1,\ldots ,N_{s}-1\\ \text{where}\;\alpha (k)=\{\begin{array}{ll} \sqrt{\frac{1}{N_{s}}} & \text{for}\;k=0\\ \sqrt{\frac{2}{N_{s}}} & \text{for}\;k\neq 0 \end{array} \end{array}$$

In these equations, *s_i_* is the *i*th element of the discrete-time sequence s, *E*{.} denotes the expectation operator, *μ***_s_** and *σ* are the mean and the standard deviation of s, *R*_ss_(Δ) is the unbiased autocorrelation sequence of **s**, *μ***_u_** is the mean of **u**, *R***_su_**(Δ) is the unbiased cross-correlation sequence between **s** and **u**, *S*_DFT_(*k*) and *S*_DCT_(*k*) are the *k*th elements of the 1-D *N_s_*-point DFT and *N_s_*-point DCT, respectively.

In constructing the feature vectors based on the acquired signals, features of the two gyroscope signals that correspond to the same 10-second time window are included in each feature vector. A total of 101 features are extracted from the signals of the two gyroscopes so that the size of each feature vector is 101 × 1. For each leg motion, 56 (= 7 × 8) such feature vectors are obtained. The initial set of features is as follows:

1 mean value of gyro 1 signal
2 mean value of gyro 2 signal
3 kurtosis of gyro 1 signal
4 kurtosis of gyro 2 signal
5 skewness of gyro 1 signal
6 skewness of gyro 2 signal
7 minimum value of gyro 1 signal
8 minimum value of gyro 2 signal
9 maximum value of gyro 1 signal
10 maximum value of gyro 2 signal
11 minimum value of cross-correlation between gyro 1 and gyro 2 signals
12 maximum value of cross-correlation between gyro 1 and gyro 2 signals
13-17 maximum 5 peaks of DFT of gyro 1 signal
18-22 maximum 5 peaks of DFT of gyro 2 signal
23-27 the 5 frequencies corresponding to the maximum 5 peaks of DFT of gyro 1 signal
28-32 the 5 frequencies corresponding to the maximum 5 peaks of DFT of gyro 2 signal
33-38 6 samples of the autocorrelation function of gyro 1 signal (sample at the midpoint and every 25th sample up to the 125th)
39-44 6 samples of the autocorrelation function of gyro 2 signal (sample at the midpoint and every 25th sample up to the 125th)
45 minimum value of the autocorrelation function of gyro 1 signal
46 minimum value of the autocorrelation function of gyro 2 signal
47-61 15 samples of the cross-correlation between gyro 1 and gyro 2 signals (every 20th sample)
63-81 first 20 DCT coefficients of gyro 1
82-101 first 20 DCT coefficients of gyro 2

Because the initial set of features was quite large (101) and not all features were equally useful in discriminating the motions, we reduced the number of features in several different ways: First, we reduced the number of features from 101 to 14 by inspection, trying to identify the features that could correctly classify the motions by trial and error. These features are listed on the left in Table 1. Then, by applying Principal Component Analysis (PCA) (see the Appendix) to these 14 selected features, we further reduced their number to six. Third, we selected the 14 features with the largest variances using the covariance matrix of the feature vectors. These features are listed on the right in Table 1. We also reduced the 101 features to eight through PCA. The features selected by PCA correspond to linear combinations of the initial set of features, obtained through a matrix transformation. Since physical meaning cannot be assigned to these features, we do not list them here.

Finally, we employed the sequential forward feature selection (SFFS) algorithm [57]. As opposed to feature reduction methods such as PCA, feature selection methods use the extracted features themselves that do have physical meaning. In SFFS, starting with a null feature set, features are added one at a time to the feature set such that with each addition, the correct classification rate is maximized. Adding new features is terminated when the desired correct classification rate or the maximum number of allowed features is reached. In our study, the techniques summarized in Section 4. are used for motion classification. We primarily use the arithmetic average of the correct classification rates obtained with these techniques as a guideline to ultimately determine the reduced feature set, although the individual performances of the different classification techniques are also considered.

The average correct classification rate with each newly added feature is given in Table 2 for two separate runs of the algorithm. As a result, the following features are selected in the given order:

1. maximum value of gyro 1 signal
2. maximum value of the cross-correlation between gyro 1 and gyro 2 signals
3. minimum value of gyro 2 signal
4. the 3rd maximum peak of DFT of gyro 2 signal
5. minimum value of the cross-correlation between gyro 1 and gyro 2 signals
6. the 3rd maximum peak of DFT of gyro 1 signal

**Figure 6.**
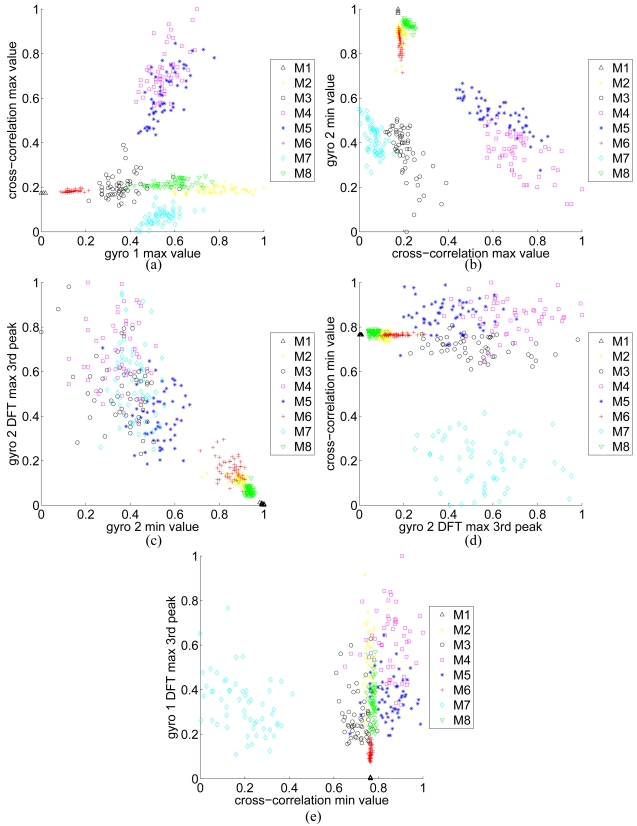
Scatter plots of the features selected by SFFS.

All of these features are normalized to the interval [0, 1] to be used for classification. Scatter plots of these features are given in Figure 6 pairwise, in the order that they have been selected. As expected, in the first two plots or so (parts (a) and (b) of the figure), the features for different classes are better clustered and more distinct.

We assume that after feature reduction or selection, the resulting feature vector is an *N* × 1 vector **x** = [*x*_1_, …, *x_N_*]*^T^*.

## 4. Classification Techniques

We associate a class *ω_i_* with each motion type (*i* = 1, …, *c*). An unknown motion is assigned to class *ω_i_* if its feature vector **x** = [*x*_1_, …, *x_N_*]*^T^* falls in the region Ω*_i_* A rule that partitions the decision space into regions Ω*_i_, i* = 1, …, *c* is called a *decision rule*. In our work, each one of these regions corresponds to a different motion type. Boundaries between these regions are called *decision surfaces*. The *training set* contains a total of *I* = *I*_1_ + *I*_2_ + … + *I_c_* sample feature vectors where *I_i_* sample feature vectors belong to class *ω_i_*, and *i* = 1, …, *c*. The *test set* is then used to evaluate the performance of the decision rule.

### 4.1. Bayesian Decision Making (BDM)

In this method, the maximum a posteriori (MAP) decision rule is used for classification. Let *p*(*ω_i_*) be the a priori probability of the motion belonging to class *ω_i_*. To classify a motion with feature vector **x**, a posteriori probabilities *p*(*ω_i_*∣**x**) are compared and the motion is classified into class *ω_j_* that has the maximum a posteriori probability such that *p*(*ω_j_*∣**x**) > *p*(*ω_i_*∣**x**) ∀*i* ≠ *j*. This is known as *Bayes' minimum error rule* and can be equivalently expressed as:

(2) $$\ell (\text{x})=\arg\;\underset{j}{\max}p(\omega_{j}|\text{x})$$

where *ℓ*(**x**) denotes the label for feature vector **x**. However, because these a posteriori probabilities are rarely known, they need to be estimated. A more convenient formulation of this rule can be obtained by using Bayes' theorem:

(3) $$p(\omega_{i}|\text{x})=\frac{p(\text{x}|\omega_{i})p(\omega_{i})}{p(\text{x})}$$

where *p*(**x**∣*ω_i_*) are the class-conditional probability density functions (CCPDFs) which are also unknown and need to be estimated in their turn based on the training set. In Equation (3), $p(\text{x})=\sum_{i=1}^{c}p(\text{x}|\omega_{i})p(\omega_{i})$ is a constant and is equal to the same value for all classes. Then, the decision rule becomes: if *p*(**x**∣*ω_j_*)*p*(*ω_j_*) > *p*(**x**∣*ω_i_*)*p*(*ω_i_*) ∀*i* ≠ *j* ⇒ **x** ∈ Ω*_j_*.

In addition, if the a priori probabilities *p*(*ω_i_*) are assumed to be equal for each class, the a posteriori probability becomes directly proportional to the likelihood value *p*(**x**∣*ω_i_*). Under this assumption, the decision rule simplifies to:

(4) $$\ell (\text{x})=\arg\;\underset{j}{\max}p(\text{x}|\omega_{j})$$

The decision rule can be generalized as *q_j_*(**x**) > *q_i_*(**x**) ∀*i* ≠ *j* ⇒ **x** ∈ Ω*_j_*, where the function *q_i_* is called a *discriminant function*.

The various statistical techniques for estimating the CCPDFs based on the training set are often categorized as non-parametric and parametric. In non-parametric methods, no assumptions on the parametric form of the CCPDFs are made; however, this requires large training sets because any non-parametric PDF estimate based on a finite number of samples is biased [58]. In parametric methods, specific models for the CCPDFs are assumed and then the parameters of these models are estimated. Parametric methods can be further categorized as normal and non-normal models.

In our study, the CCPDFs are assumed to have normal or Gaussian parametric form, and the parameters of the Gaussian distribution are estimated using maximum likelihood (ML) estimators. For class *i*, suppose a set of training vectors is given as {**x***_i_*_1_, …, **x***_iIi_*}. Then the ML estimates for the mean vector and the covariance matrix are

(5) $${\hat{\mu }}_{i}=\frac{1}{I_{i}}\sum_{j=1}^{I_{i}}{\text{x}}_{ij}$$

(6) $${\hat{\Sigma }}_{i}=\frac{1}{I_{i}}\sum_{j=1}^{I_{i}}({\text{x}}_{ij}-{\hat{\mu }}_{i}){\left({\text{x}}_{ij}-{\hat{\mu }}_{i}\right)}^{T}$$

Note that the ML estimator of the covariance is biased. Using these estimates, the CCPDF for class *i* is given as

(7) $$p(\text{x}|\omega_{i})=\frac{1}{{\left(2\pi \right)}^{\left(I_{i}/2\right)}|{\hat{\Sigma }}_{i}{|}^{\left(1/2\right)}}\exp\left\{-\frac{1}{2}{\left(\text{x}-{\hat{\mu }}_{i}\right)}^{T}{\hat{\Sigma }}_{i}^{-1}(\text{x}-{\hat{\mu }}_{i})\right\}$$

Using Equations (5)–(7), the CCPDFs are estimated for each class. Then, for a given test vector **x**, the decision rule in Equation (4) is used for classification.

### 4.2. Rule-Based Algorithm (RBA)

A rule-based algorithm or a decision tree can be considered a sequential procedure that classifies given inputs [59]. An RBA follows predefined rules at each node of the tree and makes binary decisions based on these rules. Rules correspond to conditions such as “is feature *x_i_* ≤ *τ_i_*?,” where *τ* is the threshold value for a given feature and *i* = 1, 2, …, *T*, with *T* being the total number of features used [60]. Selecting and calculating features before using them in the RBA is an important necessary issue to make the algorithm independent of the calculation cost of different features. These rules are determined by examining the training vectors of all classes. More discriminative features are used at the nodes higher in the tree hierarchy. Decision-tree algorithms start from the top of the tree and branch out at each node into two descendant nodes based on checking conditions similar to above. This process continues until one of the leaves is reached or until a branch is terminated.

As the information necessary to differentiate between the motions is completely embodied in the decision rules, the RBA has the advantage of not requiring storage of any reference feature vectors. The main difficulty is in designing the rules and making them independent of absolute quantities so that they will be more generally applicable.

**Figure 7.**
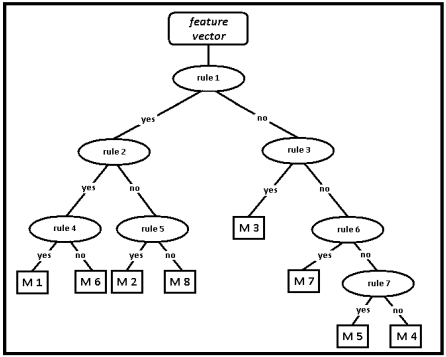
The RBA for classifying leg motions.

The RBA for the classification of leg motions has eight leaves (for the eight motions) as expected, and 7 decision nodes, as illustrated in Figure 7. These decision nodes are enumerated from top to bottom and from left to right, respectively. The rules are determined by using the normalized values of the features between 0 and 1. The rules are inequalities that compare the value of certain features or ratios of features with a constant threshold level:

1. Is the variance of gyro 2 signal < 0.1?
2. Is the variance of gyro 1 signal < 0.1?
3. Is the min value of gyro 1 signal > 0.6?
4. Is $\frac{\text{max value of gyro}\;1\;\text{signal}}{\text{min value of gyro}\;1\;\text{signal}}<0.1$?
5. Is $\frac{\text{variance of gyro}\;2\;\text{signal}}{\text{min value of autocorrelation function of gyro}\;2}>1.04$?
6. Is max value of cross-correlation function < 0.4?
7. Is $\frac{\text{max value of gyro}\;2\;\text{signal}}{\text{min value of gyro}\;2\;\text{signal}}<1.4$?

### 4.3. Least-Squares Method (LSM)

Least-squares method is one of the simplest algorithms that can be used for classification. We have implemented LSM in two different ways: In the first approach, each test feature vector is compared with each reference vector stored in the database and the test vector is assigned to the same class as the nearest reference vector. Since this approach is the same as the *k*-NN method (when *k* is selected as 1) described below, its results are not presented separately.

In the second approach, the average reference vector for each class is calculated as a representative for that particular class. Each test vector is compared with the average reference vector (instead of each individual reference vector) as follows:

(8) $$D_{i}^{2}=\sum_{n=1}^{N}{\left(x_{n}-r_{in}\right)}^{2}={\left(x_{1}-r_{i1}\right)}^{2}+\ldots +{\left(x_{N}-r_{iN}\right)}^{2}\;i=1,\ldots ,c$$

The test vector is assigned to the same class as the nearest average reference vector. In this equation, **x** = [*x*_1_, *x*_2_, …, *x_N_*]*^T^* represents a test feature vector, **r** = [*r_i_*_1_, *r_i_*_2_, …, *r_iN_*]*^T^* represents the average of the reference feature vectors for each distinct class, and $D_{i}^{2}$ is the square of the distance between these two vectors.

### 4.4. *k*-Nearest Neighbor (*k*-NN) Algorithm

Consider the *k* nearest neighbors of a feature vector **x** in a given set of many feature vectors. The neighbors are taken from a set of feature vectors (the training set) for which the correct classification is known. The occurrence number of each class is counted among these neighbor vectors and suppose that *k_i_* of these *k* vectors come from class *ω_i_*. Then, a *k*-NN estimator for class *ω_i_* can be defined as $\hat{p}(\omega_{i}|\text{x})=\frac{k_{i}}{k}$, and *p̂*(**x**∣*ω_i_*) can be obtained from *p̂*(**x**∣*ω_i_*)*p̂*(*ω_i_*) = *p̂*(*ω_i_*∣**x**)*p̂*(**x**). This results in a classification rule such that **x** is classified into class *ω_j_* if *k_j_* = max*_i_*(*k_i_*), where *i* = 1, …, *c*. In other words, the *k* nearest neighbors of the vector **x** in the training set are considered and the vector **x** is classified into the same class as the majority of its *k* nearest neighbors [61]. It is common to use the Euclidean distance measure, although other distance measures such as the Manhattan distance could in principle be used instead. The *k*-NN algorithm is sensitive to the local structure of the data.

Assigning training feature vectors to predefined classes and storing them for distance comparison can be thought of as the training phase of this technique, although no explicit training step is required. Calculating the distances of test vectors to each of the training vectors and selecting those with the *k* smallest distances comprises the test phase.

**Figure 8.**
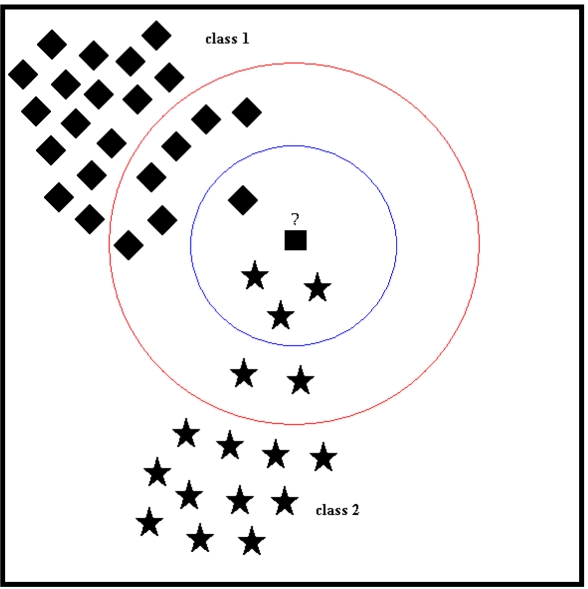
An example of the selection of the parameter *k* in the *k*-NN algorithm. The inner circle corresponds to *k* = 4 and the outer circle corresponds to *k* = 12, producing different classification results for the test vector.

For example in Figure 8, suppose that the square is the test vector and the diamonds and stars are the vectors that come from two different classes, class 1 and class 2, respectively. If *k* = 4, the four vectors in the inner circle are then the nearest neighbors of the test vector (square). Three of these vectors belong to class 2 and the remaining one belongs to class 1, so the test vector will be classified as a class 2 vector. If *k* = 12, then the classes of the nearest 12 vectors should be inspected to determine the classification of the test vector (square). These 12 vectors can be seen inside the larger circle in Figure 8. Seven of these vectors are represented with diamonds (class 1) and the remaining five are stars (class 2), so the test vector (square) will be classified as a class 1 vector. As can be seen from the above example, selection of the parameter *k*, the number of neighbors considered, is a very important issue that can affect the classification decision. Unfortunately, a pre-defined rule for selecting the value of *k* does not exist [62]. In this study, the number of nearest neighbors *k* is determined by maximizing the correct classification rate over different *k* values. When *k* = 1, the feature vector is simply assigned to the class of its nearest neighbor and this is, in fact, same as the first approach used in LSM.

### 4.5. Dynamic Time Warping (DTW)

Dynamic time warping is an algorithm for measuring the similarity between two sequences that may vary in time or speed. An optimal match between two given sequences (e.g., a time series) is found under certain restrictions. The sequences are “warped” non-linearly in the time dimension to determine a measure of their similarity independent of certain non-linear variations in the time dimension. DTW is used mostly in finite vocabulary speech recognition to handle different speaking speeds [63, 64]. Besides speech recognition, DTW has been used for word spotting in handwritten historical documents on electronic media [65] and machine-printed documents [66], in signature [67, 68] and gait recognition [69], for ECG signal classification [70–72], for fingerprint verification [73], and for face localization in color images [74]. In this study, DTW is used for classifying feature vectors extracted from gyroscope signals.

In DTW, the aim is to find the least-cost warping path for the tested feature vector among the stored reference feature vectors [63]. The cost measure is typically taken as the Euclidean distance between the elements of the feature vectors. Given two feature vectors **x** and **y** with lengths *N* and *M*:

(9) $$\begin{array}{l} \text{x}={\left[x_{1},x_{2},\ldots ,x_{n},\ldots ,x_{N}\right]}^{T}\\ \text{y}={\left[y_{1},y_{2},\ldots ,y_{m},\ldots ,y_{M}\right]}^{T} \end{array}$$

an *N* × *M* distance matrix **d** is constructed by using all the elements of the feature vectors **x** and **y**. The (*n, m*)th element of this matrix, *d*(*n, m*), is the distance between the *n*th element of **x** and the *m*th element of **y** and is given by $d(n,m)=\sqrt{{\left(x_{n}-y_{m}\right)}^{2}}=\;|x_{n}-y_{m}|$ [64].

A warping path **W** is a contiguous set of matrix elements that defines a mapping between **x** and **y**. Assuming that the *l*th element of the warping path is *w_l_* = (*n_l_, m_l_*), the warping path **W** with length *L* is given as:

(10) $$\text{W}=w_{1},w_{2},\ldots ,w_{l},\ldots ,w_{L}\;\max(N,M)\leq L<N+M-1$$

The minimum length of the warping path corresponds to max *(N, M)*, corresponding to the length of the diagonal of d when *N = M*. The maximum length is *L = N + M* − 1 when the warping path follows the two edges of the distance matrix.

The warping path W must minimize the overall cost function

(11) $$C(\text{W})=\min\left(\sum_{l=1}^{L}C[w_{l}]\right)=\min\left(\sum_{l=1}^{L}d(n_{l},m_{l})\right)$$

with the following four conditions [63, 64, 75]:

1. (monotonicity) Warping function should be monotonic, meaning that the warping function cannot go “south” or “west”:
  *n_l_ ≥ n_l_*_−1_ and *m_l_ ≥ m_l_*_−1_
2. (boundary condition) The two vectors/sequences that are compared should be matched at the beginning and the end points of the warping path:
  *w*_1_ = (1, 1) and *w_L_* = (*N, M*)
3. (continuity condition) Warping function should not bypass any points:
  *n_l_* − *n_l_*_−1_ ≤ 1 and *m_l_* − *m_l_*_−1_ ≤ 1
4. Maximum amount of warp is controlled by a global limit:
  |*n_l_* − *m_l_*| *<G*
  This global constraint *G* is called a *window width* and is used to speed up DTW and prevent pathological warpings [64]. A good path is unlikely to wander very far from the diagonal.

For a given pair of sequences, many different warping paths between (1, 1) and (*N, M*) exist but the aim is to find the least-cost one. Therefore, a cumulative distance or cost matrix **D** is constructed starting at (*n, m*) = (1, 1). *D*(*n, m*) represents the cost of the least-cost path that can be obtained until reaching point (*n, m*). As stated above, the warp path must either be incremented by one or stay the same along the *n* and *m* axes. Therefore, the distances of the optimal warp paths one data point smaller than lengths *n* and *m* are contained in the matrix elements *D*(*n* − 1, *m* − 1),*D*(*n* − 1, *m*), and *D*(*n, m* − 1). Therefore, *D*(*n, m*) is calculated by:

(12) D(n,m)=d(n,m)+min[D(n−1,m−1),D(n−1,m),D(n,m−1)]

**Figure 9.**
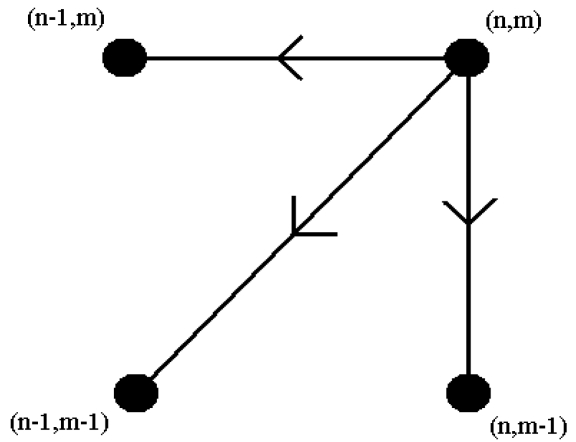
Three possible directions for constructing each step of the path.

This equation defines the cumulative distance *D*(*n, m*) as the distance *d*(*n, m*) found in the current cell and the minimum of the cumulative distances of the three adjacent cells. Because this recurrence equation determines the value of a cell by using the values in three adjacent cells, the order that the cell values are evaluated in is important: The cost matrix is filled one column at a time from the bottom up, and from left to right. The final value *D*(*N, M*) is used as a measure of distance when comparing two given feature vectors. After the entire matrix is filled, the least-cost warping path between *D*(1, 1) and *D*(*N, M*) can be found if needed. This can be calculated very efficiently by using dynamic programming, starting with the (*N, M*) element and going backwards until reaching (1, 1). At each step, adjacent cells at the left, at the bottom, and at the lower-left diagonal of the present cell are checked. In Figure 9, the three possible directions for constructing each step of the path are illustrated. Whichever of these three cells has the smallest value is added to the warp path found so far, and the search continues from that cell. In finding the smallest value among *D*(*n* − 1, *m* − 1), *D*(*n* − 1, *m*), and *D*(*n, m* − 1), if any two or three of these elements including *D*(*n* − 1, *m* − 1) are equal, *D*(*n* − 1, *m* − 1) is selected as the minimum. In other words, the diagonal path segment is preferred whenever possible. If *D*(*n* − 1, *m*), and *D*(*n, m* − 1) are equal and smaller than *D*(*n* − 1, *m* − 1), then *D*(*n* − 1, *m*) or *D*(*n, m* − 1) is chosen randomly. The search stops when *D*(1, 1) is reached. The rationale for using a dynamic programming approach in this problem is that instead of attempting to solve the problem all at once, solutions to sub-problems (portions of the two sequences) are found and used to iteratively find solutions until the solution is found for the entire sequence.

**Figure 10.**
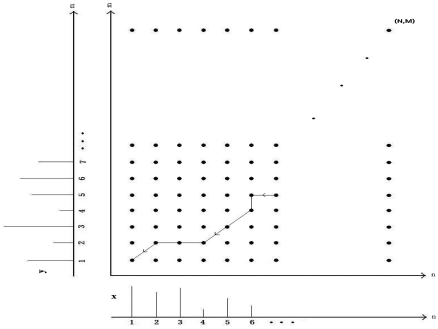
DTW mapping function.

An example warping path **W** is shown in Figure 10. Part of the DTW path in this figure is given by:

(13) W=(1,1),(2,2),(3,2),(4,2),(5,3),(6,4),(6,5),(7,5),…,(N,M)

The time and space complexity of the DTW algorithm is *O*(*NM*).

**Figure 11.**
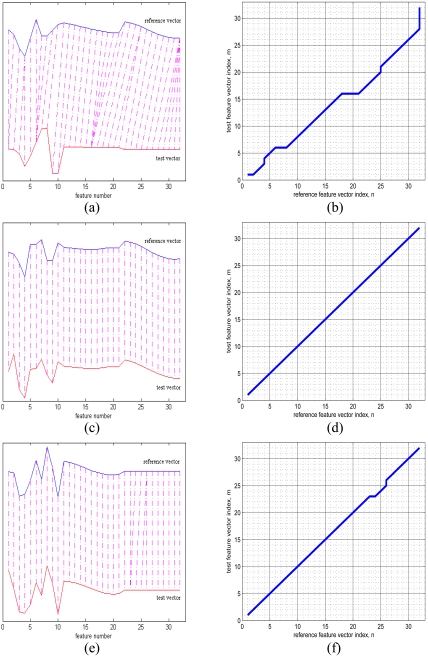
In (a), (c), and (e), the upper curves show reference vectors and the lower curves represent test vectors of size 32 × 1. Parts (b), (d), and (f) illustrate the least-cost warp paths between the two feature vectors, respectively. In (a), reference and test vectors are from different classes. In (c) and (e), both the reference and the test vectors are from the same class.

As an example, in Figure 11(a), the upper and lower curves represent a 32 × 1 reference vector and a 32 × 1 test vector from two different classes. The alignment between the samples of these two vectors is illustrated with dot-dash lines. Because these two feature vectors are very different, there is a lot of warping when they try to align, as illustrated in Figure 11(b). The reference and test vectors in part (c) of the figure both belong to the same class. Because these two vectors are very similar, warping is not observed between these two vectors, and the corresponding minimum-distance warp path shown in Figure 11(d) is a straight line. In Figures 11(e) and (f), although both the reference and the test vector belong to the same class, there appears to be a small amount of warping. As in this example, warping can sometimes occur even between reference and test vectors from the same class, resulting in classification errors.

### 4.6. Support Vector Machines (SVMs)

The support vector machine classifier is a machine learning technique proposed early in the 1980s [76, 77]. It has been mostly used in applications such as object, voice, and handwritten character recognition, and text classification.

If the feature vectors in the original feature space are not linearly separable, SVMs pre-process and represent them in a space of higher dimension where they can become linearly separable. The dimension of the transformed space may sometimes be much higher than the original feature space. With a suitable nonlinear mapping *ϕ*(.) to a sufficiently high dimension, data from two different classes can always be made linearly separable, and separated by a hyperplane. The choice of the nonlinear mapping depends on the prior information available to the designer. If such information is not available, one might choose to use polynomials, Gaussians, or other types of basis functions. The dimensionality of the mapped space can be arbitrarily high. However, in practice, it may be limited by computational resources. The complexity of SVMs is related to the number of resulting support vectors rather than the high dimensionality of the transformed space.

Consider SVMs in a binary classification setting. We are given the training feature vectors **x***_i_* that are vectors in some space *X* ⊆ ℛ*^N^* and their labels *ℓ_i_* ∈ {−1,1} where *ℓ_i_* = *ℓ*(**x***_i_*) and *i* = 1, …, *I*. Here, *ℓ_i_* is used to label the class of the feature vectors as before. If the feature vector is a class 1 vector, then *ℓ_i_* = +1; if it is a class 2 vector *ℓ_i_* = −1. The goal in training a SVM is to find the separating hyperplane with the largest margin so that the generalization of the classifier is better. All vectors lying on one side of the hyperplane are labeled as +1, and all vectors lying on the other side are labeled as −1. The support vectors are the (transformed) training patterns that lie closest to the hyperplane and are at equal distance from it. They correspond to the training samples that define the optimal separating hyperplane and are the most difficult patterns to classify, yet the most informative for the classification task.

More generally, SVMs allow one to project the original training data in space *X* to a higher-dimensional feature space ℱ via a Mercer kernel operator *K* [78]. We consider a set of classifiers of the form $f(\text{x})=\sum_{i=1}^{I}\beta_{i}\;K(\text{x},{\text{x}}_{i})$. When *f*(**x**) ≥ 0, we label **x** as +1, otherwise as −1. When *K* satisfies Mercer's condition, *K*(**u, v**) = *ϕ*(**u**) · *ϕ*(**v**) where *ϕ*(.) : *X* → ℱ is a nonlinear mapping and “·” denotes the inner or dot product. We can then rewrite *f*(**x**) in the transformed space as *f*(**x**) = a · *ϕ*(**x**). The linear discriminant function *f*(**x**) is based on the hyperplane a · *ϕ*(**x**) = 0 where $\text{a}=\sum_{i=1}^{I}\beta_{i}\phi ({\text{x}}_{i})$ is a weight vector. Thus, by using *K*, the training data is projected into a new feature space ℱ which is often higher dimensional. The SVM then computes the *β_i_*'s that correspond to the maximal margin hyperplane in ℱ. By choosing different kernel functions, we can project the training data from *X* into spaces ℱ for which hyperplanes in ℱ correspond to more complex decision boundaries in the original space *X*. Hence, by nonlinear mapping of the original training patterns into other spaces, decision functions can be found using a linear algorithm in the transformed space by only computing the kernel *K*(**x, x***_i_*).

**Figure 12.**
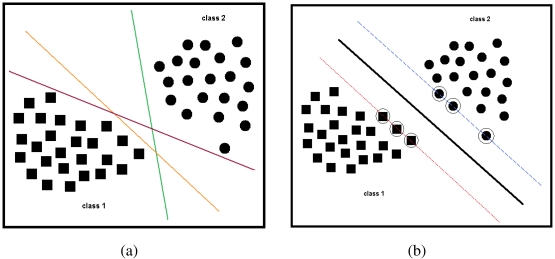
(a) Three different hyperplanes separating two classes; (b) SVM hyperplane (solid line), its margins (dotted and dashed lines), and the support vectors (circled solid squares and dots).

To illustrate the problem in 2-D, consider the training set feature vectors in Figure 12. In this example, there are two classes; squares (*ℓ_i_* = +1) symbolize the first class (class 1) and circles (*ℓ_i_* = −1) symbolize the second class (class 2). These two types of training vectors can be separated with infinitely many different hyperplanes, three of which are shown in Figure 12(a). For each of these hyperplanes, correct classification rates may be different when test vectors are presented to the system. To have the smallest classification error at the test stage, the hyperplane should be placed between the support vectors of two classes with maximum and equal margin for both classes [79]. For a SVM, the optimal hyperplane classifier is unique [60]. The equation of a hyperplane that may be used to classify these two classes is given by:

(14) a·ϕ(x)=0

and is represented by the solid line in Figure 12(b). Here, both the weight vector **a** and the transformed feature vector *ϕ*(**x***_i_*) have been augmented by one dimension to include a bias weight so that the hyperplanes need not pass through the origin. For this hyperplane to have maximum margins, dotted and dashed margin lines in Figure 12(b) are given by the following two equations, respectively:

(15) $$\begin{array}{l} \text{a}\cdot \phi (\text{x})=1\\ \text{a}\cdot \phi (\text{x})=-1 \end{array}$$

In the same figure, vectors that are marked with extra circles correspond to the support vectors.

Because there should not be training set vectors dropping between these margin lines, the following equations should be satisfied:

(16) $$\begin{array}{l} \text{a}\cdot \phi ({\text{x}}_{i})\geq 1,\forall {\text{x}}_{i}\in \text{class}\;1\\ \text{a}\cdot \phi ({\text{x}}_{i})\leq -1,\forall {\text{x}}_{i}\in \text{class}\;2 \end{array}$$

More compactly, a separating hyperplane ensures

(17) $$\ell_{i}f({\text{x}}_{i})=\ell_{i}\text{a}\cdot \phi ({\text{x}}_{i})\geq 1\;\text{for}\;i=1,\ldots ,I$$

Assuming a = [**n**, *a*_0_] where **n** is the normal vector of the hyperplane, it can be shown that the distance between the two margin lines is 2/‖**n**‖. Therefore, to maximize the separation between these margin lines, ‖**n**‖ should be minimized. Since *a*_0_ is a constant, this is equivalent to minimizing ‖**a**‖.

To have optimal margin hyperplanes for classifying feature vectors, the optimal hyperplane can be found by minimizing the magnitude of the weight vector ‖ **a** ‖^2^ subject to the constraint given by Equation (17) [80]. Using the method of Lagrange multipliers, we construct the functional

(18) $$ℒ(\text{a},\lambda )=\frac{1}{2}{\left\|\text{a}\right\|}^{2}-\sum_{i=1}^{I}\lambda_{i}[\ell_{i}\text{a}\cdot \phi ({\text{x}}_{i})-1]$$

where the second term in the above equation expresses the goal of classifying the points correctly. To find the optimal hyperplane, we minimize ℒ(.) with respect to the weight vector **a**, while maximizing with respect to the undetermined Lagrange multipliers *λ_i_* ≥ 0. This can be done by solving the constrained optimization problem by quadratic programming [81] or by other techniques. The solution of the weight vector is ${\text{a}}^{*}=\sum_{i=1}^{I}\ell_{i}\lambda_{i}\phi ({\text{x}}_{i})$, corresponding to *β_i_* = *ℓ_i_λ_i_*. Then, the decision function is given by:

(19) $$f^{*}(\text{x})=\sum_{i=1}^{I}\lambda_{i}\ell_{i}\phi ({\text{x}}_{i})\cdot \phi (\text{x})$$

In this study, the method summarized above is applied to differentiate feature vectors that belong to more than two classes. Following the one-versus-the-rest method, *c* different binary classifiers are trained, where each classifier recognizes one of *c* motion types.

In this study, the performance of linear classifiers was not satisfactory for classifying human leg motions. Therefore, a nonlinear classifier is used with a radial basis function (RBF) kernel according to the following model with *γ* = 4:

(20) $$K(\text{x},{\text{x}}_{i})=e^{-\gamma |\text{x}-{\text{x}}_{i}{|}^{2}}$$

A library for SVMs (LIBSVM toolbox) is used in the MATLAB environment [82].

### 4.7. Artificial Neural Networks (ANN)

Multi-layer ANNs consist of an input layer, one or more hidden layers to extract progressively more meaningful features, and a single output layer, each comprised of a number of units called *neurons*. The model of each neuron includes a smooth nonlinearity, which is called the *activation function*. Due to the presence of distributed nonlinearity and a high degree of connectivity, theoretical analysis of ANNs is difficult. These networks are trained to compute the boundaries of decision regions in the form of connection weights and biases by using training algorithms. The performance of ANNs is affected by the choice of parameters related to the network structure, training algorithm, and input signals, as well as by parameter initialization [83, 84].

In this work, a three-layer ANN is used for classifying leg motions. The input layer has *N* neurons, equal to the dimension of the feature vectors. The hidden layer has nine neurons, and the output layer has *c* neurons, equal to the number of classes. In the input and hidden layers each, there is an additional neuron with a bias value of 1. For an input feature vector **x** ∈ ℝ*^N^*, the target output is 1 for the class that the vector belongs to, and 0 for all other output neurons. The sigmoid function used as the activation function in the hidden and output layers is given by:

(21) $$g(x)=\frac{1}{1+e^{-x}}$$

The output neurons can take continuous values between 0 and 1. Fully-connected ANNs are trained with the back-propagation algorithm (BPA) [83] by presenting a set of *training patterns* to the network. Different initial conditions and different numbers of neurons in the hidden layer have been considered.

The aim is to minimize the average of the sum of squared errors over all training vectors:

(22) $${ℰ}_{\text{av}}(\text{w})=\frac{1}{2I}\sum_{i=1}^{I}\sum_{k=1}^{c}{\left[t_{ik}-o_{ik}(\text{w})\right]}^{2}$$

Here, **w** is the weight vector, *t_ik_* and *o_ik_* are the desired and actual output values for the *i*th training pattern and the *k*th output neuron, and *I* is the total number of training patterns. When the entire training set is covered, an *epoch* is completed. The error between the desired and actual outputs is computed at the end of each iteration and these errors are averaged at the end of each epoch (Equation (22)). The training process is terminated when a certain precision goal on the average error is reached or if the specified maximum number of epochs (5,000) is exceeded, whichever occurs earlier. The latter case occurs very rarely. The acceptable average error level is set to a value of 0.06. The weights are initialized randomly with a uniform distribution in the interval [0,1], and the learning rate is chosen as 0.3.

In the test phase, the test feature vectors are fed forward to the network, the outputs are compared with the desired outputs and the error between them is calculated. The test vector is said to be correctly classified if this error is below a threshold value of 0.15.

## 5. Experimental Results

In this study, the classification techniques described in the previous section are used to classify eight different leg motions. A total of 448 (= 7 × 8 × 8) feature vectors are available. In the training and testing phases of the classification process, we use the following approaches: repeated random sub-sampling (RRSS), *P*-fold, and leave-one-out (LOO) cross-validation techniques. In RRSS, we divide the 56 feature vectors from each motion type randomly into two sets so that each set contains 28 feature vectors. In total, 224 (= 28 × 8) vectors are used for training and the same number of vectors is used for testing. This is repeated 100 times and the resulting correct differentiation percentages are averaged. The disadvantage of this method is that because of the randomization, some feature vectors may never be selected in the testing or the validation phase, whereas others may be selected more than once. In other words, validation subsets may overlap.

In *P*-fold cross validation, the 448 feature vectors are divided into *P* = 8 partitions, where each partition contains seven randomly selected feature vectors from each class, for a total of 56 vectors. Of the *P* partitions, a single partition is retained as the validation set for testing, and the remaining *P* − 1 partitions are used for training. The cross-validation process is then repeated *P* times (the folds), where each of the *P* partitions is used exactly once for validation. The *P* results from the folds are then averaged to produce a single estimation. This process is repeated 100 times and the average correct differentiation percentage is reported. The advantage of this validation method over RRSS is that all feature vectors are used for both training and testing, and each feature vector is used for testing exactly once in each of the 100 runs.

Finally, we also used LOO cross validation, where a single feature vector out of 448 is used in turn for validation, and the remaining 447 feature vectors are used for training. This is repeated such that each feature vector is used once as the validation data (i.e., 448 times) and the correct classification rate is calculated. This is the same as a *P*-fold cross validation with *P* being equal to the number of feature vectors in the original sample (*P* = 448). Because the training process is repeated a large number of times, the LOO cross-validation technique is often computationally expensive.

Correct differentiation rates obtained with different classification techniques are given in Tables 3–5 for the five different feature sets we considered and the three different cross-validation techniques. For the RBA, the features used in the rules do not correspond to any of the sets presented in Tables 3–5. Therefore, RBA results are not listed in these tables. Correct differentiation rates of 95.2%, 95.1%, and 95.1% are achieved with RBA for RRSS, *P*-fold, and LOO cross-validation techniques, respectively.

Among the five different feature sets that we considered, the first two and the last one result in higher classification rates in general. As the last feature set (obtained by SFFS) can be obtained more systematically, we used this feature set in reporting the confusion matrices of the different techniques.

From the tables, it can be observed that there is not a significant difference between the results of different cross-validation techniques in terms of their classification accuracy. Among the classification techniques we have considered and implemented, BDM in general gives the highest classification rate, followed by SVM, with a few exceptions. As LOO cross validation gives slightly larger correct differentiation rates, this cross-validation technique is used in obtaining the confusion matrices of the classification techniques presented in Tables 6–12. Looking at the confusion matrices of the different techniques, it can be observed that motions 4 and 5 and motions 2 and 8 are the motions mostly confused with each other. Motions 4 and 5 are similar in that both the lower and upper parts of the leg are moving without bending the knee forward and backward, respectively. The confusion of motions 2 and 8 is also caused by their similarity because only the lower part of the leg is moving backward and forward in these motions, respectively.

The confusion matrices for BDM and RBA are provided in Tables 6 and 7. With these methods, correct differentiation rates of 98.2% and 95.1% are, respectively, achieved.

In the LSM approach, test vectors are compared with the average of the reference vectors that are calculated for each of the eight classes. The confusion matrix for this method is provided in Table 8. The overall successful differentiation rate of LSM is 94.2%.

**Figure 13.**
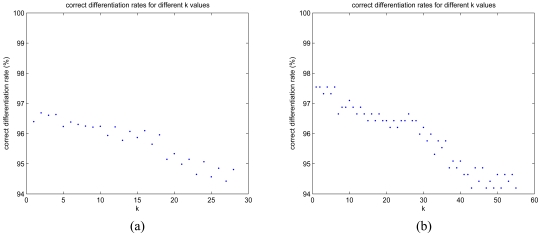
Correct classification rates of the *k*-NN algorithm for (a) *k* = 1, …, 28 (RRSS) and (b) *k* = 1, …, 55 (LOO).

Performance of the *k*-NN algorithm changes for different values of *k*. Correct differentiation rates for different *k* values are presented in Figures 13(a) and (b) for RRSS and LOO cross-validation techniques. As the value of *k* increases, the rate of successful classification decreases. Values of *k* between 1 and 4 seem to be more suitable because they provide larger classification rates. The confusion matrix of the *k*-NN algorithm for *k* = 1 is provided in Table 9, and a successful differentiation rate of 97.6% is achieved.

We implemented the DTW algorithm in two different ways: In the first approach, the average of the reference feature vectors for each motion is used for comparison. The confusion matrix for the DTW method by using this first approach (DTW-1) is presented in Table 10, and a correct differentiation rate of 96.0% is achieved. As a second approach (DTW-2), DTW distances are calculated between the test vector and each of the (56 × 8) − 1 = 447 reference vectors from different classes. The class of the nearest reference vector is assigned as the class of the test vector. Correct classification rate of this second approach is 97.3%. The corresponding confusion matrix is given in Table 11.

In SVM, following the one-versus-the-rest method, each leg motion is assumed as the first class and the remaining seven leg motions are assumed as the second class. With LOO cross validation, a different SVM model is created for the classification of each test vector. Since there are 56 test vectors for each motion type, a total of 448 different SVM models are created. The number of correctly and incorrectly classified feature vectors for each motion type is tabulated in Table 12(a). The overall correct classification rate of the SVM method is calculated as 98.2%.

For ANN, since the incorrectly classified feature vectors are usually classified as belonging to none of the classes, it is not possible to form a more detailed confusion matrix. The number of correctly and incorrectly classified feature vectors with LOO cross validation is given in Table 12(b). The overall correct classification rate of this method is the lowest (80.1%) among all the methods considered. On the average, the network converges in about 400 epochs for the LOO cross-validation technique.

### 5.1. Computational Cost of the Classification Techniques

The classification techniques given above are also compared based on their computational costs. Pre-processing and classification times are calculated on an Intel Centrino Duo CPU T2400 @1.83 GHz, 0.99 GB RAM laptop computer running the Microsoft Windows XP Professional operating system. Pre-processing and storage requirements of the different techniques are tabulated in Table 13. The pre-processing time of BDM is used for estimating the mean vector, covariance matrix and the CCPDFs, which need to be stored for the test stage. In RBA, the pre-processing phase involves extracting the rules based on the available data. Once the rules are available, the vectors need not be stored and any test vector can be classified using the RBA. In LSM and DTW-1, the averages of the training vectors for each class need to be stored for the test phase. Note that the pre-processing time for these two methods is exactly the same. For *k*-NN and DTW-2, all training vectors need to be stored. For the SVM, the SVM models constructed in the training phase need to be stored for the test phase. For ANN, the structure of the trained network and the connection weights need to be saved for testing. ANN and SVM require the longest training time and also have considerable storage requirements.

The resulting processing times of the different techniques for classifying a single feature vector are given in Table 14. The classification time for RBA is the smallest, followed by SVM or LSM, *k*-NN (*k* = 1) or DTW-1 or the ANN, next BDM, and last DTW-2 methods. DTW-2 takes the longest amount of classification time due to the nature of the algorithm and also because a comparison should be made with every training vector. Among the different cross-validation techniques, RRSS requires the shortest amount of processing time, whereas in general, LOO requires the longest.

### 5.2. Discussion

Given its very high correct classification rate and relatively small pre-processing and classification times and storage requirements, it can be concluded that BDM is superior to the other classification techniques we considered for the given classification problem. This result supports the idea that the distribution of the motions in the feature space can be well approximated by multi-dimensional Gaussian distributions. The low processing and storage requirements of the BDM method make it a strong candidate for similar classification problems.

The support vector machine method is also very accurate but requires a considerable amount of pre-processing time to construct the SVM models. For real-time applications, LSM and DTW-1 could also be suitable choices because they are faster than BDM at the expense of a slightly lower correct classification rate.

It can clearly be observed from the results that the ANN method performs significantly poorer than the other methods, and the required training time is much longer. This may be because the number of training vectors are limited, preventing the network from converging to a robust classifier. Increasing the number of hidden-layer neurons can improve the performance, however this would result in longer processing times.

## 6. Potential Application Areas

There are diverse applications in which the methods and algorithms presented in this paper can be utilized. In home-based rehabilitation and physical therapy, the required exercises for the patient can be monitored, and feedback can be provided to the patient or the therapist to maximize the efficiency of the therapy. Similar applications are sports training, physical education and dance, where the trainer and/or the trainee can be given feedback regarding their motions in terms of effectiveness and safety, as well as increasing the benefits of physical exercise, improving athletic performance, and most importantly, promoting health and preventing injuries. Integrating other sensing modalities such as accelerometers, heart rate and blood pressure monitors, more detailed judgment about the motions can be obtained and the efficiency of training and/or therapy can further be increased.

In a more general context, motion recognition and analysis using gyroscopes can be applied in biomechanics, ergonomics, remote monitoring of physically or mentally disabled, elderly, and children, detecting falls, sports science, animation and film making, computer games, professional simulators, virtual reality, and motion compensation and stabilization of equipment. The references for previous studies on some of these applications are given in Section 1.

## 7. Conclusions and Future Work

We have presented the results of a comparative study where features extracted from gyroscope signals are used for classifying leg motions. A number of classification techniques have been compared based on the same data set in terms of their correct differentiation rates, confusion matrices, computational costs, training and storage requirements. BDM achieves higher correct classification rates compared to the other classification techniques and has relatively small computational time and storage requirements. This parametric method can be employed in similar classification problems where it is appropriate to model the feature space with multi-dimensional Gaussian distributions. The support vector machine method is the second-best choice in terms of classification accuracy but it requires a considerable amount of pre-processing time to construct the models. For real-time applications, LSM and DTW-1 could also be considered suitable choices because they are faster than BDM at the expense of a slightly lower correct classification rate.

A number of feature extraction and reduction methods as well as different cross-validation techniques have been implemented and compared in this study. Although there is not a significant difference between the correct classification rates obtained by different cross-validation techniques, RRSS uses the shortest amount of processing time, whereas LOO requires the longest. However, the main disadvantage of RRSS is that some feature vectors may never be used for testing, whereas others may be used more than once. In *P*-fold and LOO cross validation, all feature vectors are used for both training and testing.

There are several possible future research directions that can be explored:

We plan to extend the classification of leg motions considered here into different daily activities performed in indoor and outdoor environments by a variety of subjects. An aspect of activity recognition and classification that has not been much investigated is the normalization between the way different individuals perform the same activities. Each person does a particular activity differently due to differences in body size, style, and timing. Although some approaches may be more prone to highlighting personal differences, new techniques need to be developed that involve time-warping and projections of signals and comparing their differentials.

To the best of our knowledge, optimizing the positioning, number, and type of sensors has not been much studied. Typically, some configuration, number, and modality of sensors is chosen and used without strong justification.

Detection and classification of falls using inertial sensors is another important problem that has not been sufficiently well investigated [18]. One of the reasons for this is the difficulty of designing and performing fair and realistic experiments in this area [27], and thus standard and systematic techniques for detecting and classifying falls still do not exist. In our ever-aging population, it seems imperative to develop such definitions and techniques as soon as possible [16, 17].

Fusion of information from inertial sensors and cameras can be further explored to provide robust solutions in human activity monitoring, recognition, and classification. Joint use of these two sensing modalities increases the capabilities of intelligent systems and enlarges the application potential of inertial and vision systems.

## A Principal Component Analysis (Karhunen-Loéve Transformation)

Principal Component Analysis (PCA) is a technique used in pattern recognition to reduce the size of feature vectors by eliminating the redundant features. Components of the feature vector are extracted from the acquired signals or real world data, and are transformed to a new space where they become uncorrelated [85]. Features with large variances are more discriminating so they are used to construct the transformation matrix, whereas features with small variances are considered as noise [75]. The steps of PCA are as follows [86]:

- Mean of each feature vector is calculated and subtracted.
- Covariance matrix of training feature vectors is calculated.
- Eigenvalues and eigenvectors of the covariance matrix are calculated.
- Transformation matrix is obtained by arranging the eigenvectors in descending order of their eigenvalues.
- Features are transformed to a new space where they become uncorrelated.

The diagonal elements of the covariance matrix are the variances of the features and the off-diagonal elements correspond to the correlation between the different features. The feature with the largest eigenvalue is the most discriminative feature, and the corresponding eigenvector is called the principal component of the data set. This eigenvector is placed on the first row of the transformation matrix. The transformed features do not correspond to any physically meaningful quantity [59].

## Figures and Tables

**Table 1.** Features selected by inspection (left) and the features selected by using the covariance matrix (right).

features selected by inspection:	features selected from the covariance matrix:
	
1: min value of cross-correlation	1: min value of gyro 2
2: max value of cross-correlation	2: min value of gyro 1
3: variance of gyro 2	3–8: 6 samples of the autocorrelation
4: min value of gyro 1	function of gyro 2
5: max value of gyro 1	9: 1st max peak of DFT of gyro 2
6: skewness of gyro 1	10: max value of gyro 2
7: skewness of gyro 2	11: min value of autocorrelation of gyro 2
8: mean of gyro 2	12: 3rd max peak of DFT of gyro 2
9: min value of gyro 2	13: max value of gyro 1
10–14: maximum 5 peaks of DFT of gyro 2	14: min value of cross-correlation

**Table 2.** Sample SFFS results where average correct classification rates over all classification techniques are given for two different runs.

**features selected (1st run):**	%	**features selected (2nd run):**	%
	
max value of gyro 1	56.7	max value of gyro 1	56.8
max value of cross-correlation	86.2	max value of cross-correlation	86.9
3rd max peak of DFT of gyro 2	93.8	min value of gyro 2	93.8
variance of gyro 2	95.0	3rd max peak of DFT of gyro 2	95.8
min value of cross-correlation	95.9	min value of cross-correlation	96.3
min value of gyro 2	96.1	skewness of gyro 1	97.2
skewness of gyro 1	96.8	2nd DCT coefficient of gyro 2	97.4
5th max peak of DFT of gyro 2	97.0		
6th DCT coefficient of gyro 2	97.2		

**Table 3.** Correct differentiation rates for different feature reduction methods and RRSS cross validation.

method:	correct differentiation rate (%)				
	by inspection (14 features)	PCA to 14 features (6 features)	covariance matrix (14 features)	PCA to 101 features (8 features)	SFFS (6 features)
BDM	97.5	97.7	96.2	98.0	97.3
LSM	97.0	96.9	91.8	88.5	94.6
*k*-NN (*k* = 1)	96.9	96.9	95.3	94.9	96.4
DTW-1	92.1	92.2	87.9	82.6	95.4
DTW-2	96.9	96.3	95.1	93.6	95.7
SVM	99.2	99.1	94.6	94.6	97.2
ANN	88.6	90.2	87.7	88.8	87.8

**Table 4.** Correct differentiation rates for different feature reduction methods and *P*-fold cross validation.

method:	correct differentiation rate (%)				
	by inspection (14 features)	PCA to 14 features (6 features)	covariance matrix (14 features)	PCA to 101 features (8 features)	SFFS (6 features)
BDM	98.9	98.5	98.1	99.1	98.1
LSM	97.3	97.5	92.1	89.5	94.6
*k*-NN (*k* = 1)	97.1	98.1	94.8	95.4	97.4
DTW-1	91.8	92.8	87.7	83.8	95.7
DTW-2	98.0	96.9	96.1	95.2	97.0
SVM	99.7	99.4	95.3	96.7	97.9
ANN	86.4	88.8	85.0	83.2	84.4

**Table 5.** Correct differentiation rates for different feature reduction methods and LOO cross validation.

method:	correct differentiation rate (%)				
	by inspection (14 features)	PCA to 14 features (6 features)	covariance matrix (14 features)	PCA to 101 features (8 features)	SFFS (6 features)
BDM	99.1	99.3	98.2	99.1	98.2
LSM	97.1	97.3	92.0	90.4	94.2
*k*-NN (*k* = 1)	97.1	98.2	94.6	95.1	97.6
DTW-1	91.7	93.8	88.0	83.7	96.0
DTW-2	98.2	97.8	95.2	95.1	97.3
SVM	98.9	98.4	96.4	98.4	98.2
ANN	85.1	88.8	84.8	83.3	80.1

**Table 6.** Confusion matrix for BDM (LOO cross validation, 98.2%).

		**classified**							
		M1	M2	M3	M4	M5	M6	M7	M8
**true**	M1	56	0	0	0	0	0	0	0
	M2	0	55	0	0	0	0	0	1
	M3	0	0	56	0	0	0	0	0
	M4	0	0	0	54	2	0	0	0
	M5	0	0	0	3	53	0	0	0
	M6	0	0	0	0	0	56	0	0
	M7	0	0	0	0	0	0	56	0
	M8	0	2	0	0	0	0	0	54

**Table 7.** Confusion matrix for RBA (LOO cross validation, 95.1%).

		**classified**							
		M1	M2	M3	M4	M5	M6	M7	M8
**true**	M1	56	0	0	0	0	0	0	0
	M2	0	56	0	0	0	0	0	0
	M3	0	0	49	0	0	0	7	0
	M4	0	0	0	46	10	0	0	0
	M5	0	0	0	4	52	0	0	0
	M6	0	0	0	0	0	56	0	0
	M7	0	0	1	0	0	0	55	0
	M8	0	0	0	0	0	0	0	56

**Table 8.** Confusion matrix for LSM (LOO cross validation, 94.2%).

		**classified**							
		M1	M2	M3	M4	M5	M6	M7	M8
**true**	M1	56	0	0	0	0	0	0	0
	M2	0	46	0	0	0	0	0	10
	M3	0	0	54	2	0	0	0	0
	M4	0	0	0	50	6	0	0	0
	M5	0	0	0	3	53	0	0	0
	M6	0	0	0	0	0	56	0	0
	M7	0	0	0	0	0	0	56	0
	M8	0	5	0	0	0	0	0	51

**Table 9.** Confusion matrix for the *k*-NN algorithm for *k* = 1 (LOO cross validation, 97.6%).

		**classified**							
		M1	M2	M3	M4	M5	M6	M7	M8
**true**	M1	56	0	0	0	0	0	0	0
	M2	0	52	0	0	0	0	0	4
	M3	0	0	56	0	0	0	0	0
	M4	0	0	0	52	4	0	0	0
	M5	0	0	0	2	54	0	0	0
	M6	0	0	0	0	0	56	0	0
	M7	0	0	0	0	0	0	56	0
	M8	0	1	0	0	0	0	0	55

**Table 10.** Confusion matrix for DTW-1 (LOO cross validation, 96.0%).

		**classified**							
		M1	M2	M3	M4	M5	M6	M7	M8
**true**	M1	56	0	0	0	0	0	0	0
	M2	0	49	0	0	0	2	0	5
	M3	0	0	56	0	0	0	0	0
	M4	0	0	0	52	4	0	0	0
	M5	0	0	1	3	52	0	0	0
	M6	0	0	0	0	0	56	0	0
	M7	0	0	2	0	0	0	54	0
	M8	0	1	0	0	0	0	0	55

**Table 11.** Confusion matrix for DTW-2 (LOO cross validation, 97.3%).

		**classified**							
		M1	M2	M3	M4	M5	M6	M7	M8
**true**	M1	56	0	0	0	0	0	0	0
	M2	0	54	0	0	0	0	0	2
	M3	0	0	56	0	0	0	0	0
	M4	0	0	0	53	3	0	0	0
	M5	0	0	0	5	51	0	0	0
	M6	0	0	0	0	0	56	0	0
	M7	0	0	1	0	0	0	55	0
	M8	0	1	0	0	0	0	0	55

**Table 12.** (a) Number of correctly and incorrectly classified feature vectors out of 56 for SVMs (LOO cross validation, 98.2%); (b) same for ANN (LOO cross validation, 80.1%).

		**(a)**	
		**classified**	
		correct	incorrect
**true**	M1	56	0
	M2	54	2
	M3	56	0
	M4	53	3
	M5	53	3
	M6	56	0
	M7	56	0
	M8	56	0

		**(b)**	
		**classified**	
		correct	incorrect
**true**	M1	56	0
	M2	20	36
	M3	52	4
	M4	21	35
	M5	43	13
	M6	56	0
	M7	56	0
	M8	55	1

**Table 13.** Pre-processing and training times and the storage requirements of the classification methods.

method:	pre-processing/training time (msec)			storage requirements
	RRSS	*P*-fold	LOO	
BDM	2.144	1.441	1.706	mean, covariance, CCPDF
RBA	–	–	–	rules
LSM	0.098	0.554	105.141	average of training vectors for each class
*k*-NN (*k* = 1)	–	–	–	all training vectors
DTW-1	0.098	0.554	105.141	average of training vectors for each class
DTW-2	–	–	–	all training vectors
SVM	72.933	1880.233	5843.133	SVM models
ANN	151940	145680	189100	network structure and connection weights

**Table 14.** The processing times required for classifying a single feature vector.

method:	classification time (msec)		
	RRSS	*P*-fold	LOO
BDM	2.588	1.220	8.188
RBA	0.003	0.003	0.003
LSM	0.070	0.074	0.063
*k*-NN (*k* = 1)	0.095	0.452	24.033
DTW-1	1.775	1.937	2.000
DTW-2	49.640	94.014	107.400
SVM	0.009	0.016	0.132
ANN	0.882	2.547	1.391
